# Personalizing immunosuppressive therapy: predictors of drug switches in Malaysian kidney transplant recipients

**DOI:** 10.3389/jpps.2025.14632

**Published:** 2025-09-08

**Authors:** Chiau Ling Choong, Farida Islahudin, Mohd Makmor-Bakry, Nor Asyikin Mohd Tahir, Hin-Seng Wong, Rosnawati Yahya

**Affiliations:** ^1^ Center of Quality Medicine Management, Faculty of Pharmacy, Universiti Kebangsaan Malaysia, Kuala Lumpur, Malaysia; ^2^ Faculty of Pharmacy, Universitas Airlangga, Surabaya, Indonesia; ^3^ Department of Nephrology, Selayang Hospital, Ministry of Health Malaysia, Batu Caves, Malaysia; ^4^ Sunway Medical Centre, Department of Nephrology, Subang Jaya, Malaysia; ^5^ Department of Nephrology, Kuala Lumpur Hospital, Ministry of Health Malaysia, Kuala Lumpur, Malaysia

**Keywords:** tacrolimus, everolimus, immunosuppressive agents, kidney transplantation, immunosuppression switch

## Abstract

**Objective:**

Tacrolimus-mycophenolic acid (MPA)-prednisolone immunosuppression remains the first-line management of kidney transplantation. Despite this, a switch to low-dose tacrolimus in combination with an mTOR inhibitor may be inevitable in some patients due to various factors. This study aims to identify the reasons and factors influencing the switch of tacrolimus-MPA to other combination immunosuppressive agents among kidney transplant recipients (KTRs).

**Methods:**

This retrospective observational cohort study included adult KTRs between year 2011–2019 at the two main kidney transplant centers in Malaysia. Demographic data, clinical, laboratory and medication information were collected. Multiple logistic regression was used to determine factors associated with the initial switch of tacrolimus-MPA immunosuppressive therapy.

**Results:**

From the 257 KTRs studied, 81 KTRs had their immunosuppressive agents switched from tacrolimus-MPA-prednisolone immunosuppressive regimen, with majority (96.3%, n = 78) switching to everolimus, an mTOR inhibitor in combination with low-dose tacrolimus. The average time switch was 125.8 ± 100.9 days. The main reasons for the initial switch include unresolved transaminitis (n = 15, 18.5%), cytomegalovirus (CMV) infection (n = 13, 16.0%) and BK virus (BKV) infection (n = 10, 12.3%). In the multiple logistic analysis, Malay ethnicity (P < 0.001), KTRs without post-transplant hypertension (P = 0.004) and KTRs with BKV infection (P < 0.001) were predictors for the initial switch of tacrolimus-MPA-prednisolone immunosuppressive therapy.

**Conclusion:**

Early identification of factors associated with the switch may prepare healthcare professionals for KTRs risk stratification, allowing ample time for appropriate optimization of tacrolimus-MPA-prednisolone immunosuppressive therapy based on individual patient’s needs. This can possibly be a cost-effective alternative to switching to mTOR inhibitors for improved transplant outcomes.

## Introduction

Kidney transplantation in Malaysia has evolved significantly over the past few decades and remains an important option for patients diagnosed with end stage kidney disease [1]. The number of patients on dialysis has increased exponentially while kidney transplants are limited due to lack of donors [2]. In the 13th report of the Malaysian National Transplant Registry, the number of new kidney transplant performed was 113 in 2007, however this has decreased to 82 transplant cases performed in 2016 [2]. In Malaysia, most kidney transplants are performed in two major government hospitals, with some private hospitals offering transplant services too.

Following kidney transplantation, kidney transplant recipients (KTRs) will be given a combination of immunosuppressants. Among these, the most common maintenance treatment is a combination of a calcineurin inhibitor (CNI), that is tacrolimus or cyclosporin, an antiproliferative agent namely mycophenolate mofetil (MMF) or enteric-coated mycophenolate sodium (EC-MPS) and a corticosteroid [3]. Tacrolimus is the preferred first-line CNI for long-term maintenance immunosuppression following kidney transplantation [3]. This preference stems from its demonstrably superior efficacy in reducing the incidence and severity of biopsy-proven acute rejection (BPAR) episodes, a critical complication known to significantly compromise graft function and potentially lead to graft loss [4]. Furthermore, tacrolimus exhibits several pharmacological advantages where preclinical and clinical data suggest a potentially lower risk of nephrotoxicity, a major long-term concern in KTRs with pre-existing compromised kidney function [4]. Additionally, tacrolimus boasts a more predictable pharmacokinetic profile, allowing for more precise individualization of dosing regimens [4]. This characteristic enables clinicians to optimize immunosuppressive efficacy while minimizing the risk of adverse effects through therapeutic drug monitoring, supporting its use as the gold standard CNI for maintenance immunosuppression in KTRs.

Despite demonstrably improved short-term outcomes following kidney transplantation, long-term graft function and survival rates remain stagnant beyond the first year [5]. This plateauing effect is likely attributable to the development of chronic adverse drug reactions (ADRs) associated with long-term CNI use [5]. CNIs, while effective in preventing rejection, carry a significant burden of nephrotoxicity, directly damaging the transplanted kidney and potentially leading to graft dysfunction [6]. Furthermore, CNIs are implicated in other long-term ADRs that significantly impact patient morbidity and mortality. These include post-transplant diabetes mellitus (PTDM), characterized by impaired insulin sensitivity and contributing to cardiovascular complications [6]. Additionally, CNIs’ immunosuppressive nature increases the risk of various malignancies, particularly skin cancers, necessitating vigilant monitoring as well as compromising the immune system’s ability to combat viral infections, potentially leading to serious complications like cytomegalovirus (CMV) reactivation [6]. The cumulative burden of these chronic ADRs necessitates exploring alternative immunosuppressive strategies or minimizing CNI use as early as 3 months post-transplant in some patients [7]. This ongoing challenge underscores the need for novel immunosuppressive approaches that balance efficacy with a minimized risk of long-term complications to optimize long-term graft function and patient outcomes.

In the quest to mitigate the long-term complications, such as adverse effects and toxicities associated with tacrolimus-based immunosuppression regimens in kidney transplantation, switching to the mammalian target of rapamycin (mTOR) inhibitors like everolimus have emerged as a promising strategy. Studies have demonstrated improved overall graft function following a switch to everolimus combined with reduced-dose tacrolimus, without compromising the rates of BPAR or viral infections [8, 9]. However, this approach is not without drawbacks. mTOR inhibitors themselves carry a risk of ADRs including metabolic complications and even acute rejection episodes [8]. Additionally, the higher cost of these agents and the need for frequent laboratory monitoring in the initial period following regimen switch present significant economic and logistical considerations [10]. Therefore, the optimal balance between minimizing tacrolimus-related toxicities and mitigating the potential ADRs of mTOR inhibitors remains an ongoing area of research in optimizing long-term outcomes for KTRs.

This study aims to identify factors predictive of switching from the tacrolimus-MPA immunosuppressive regimen to alternative combinations in KTRs. Early identification of patients at high risk for regimen switches would enable healthcare professionals to implement closer monitoring and potentially optimize CNI-based immunosuppression according to individual patient’s needs, thereby minimizing complications associated with chronic ADRs. To date, no prior research, be it local, regional or international, has explored the factors influencing the initial switch from tacrolimus-MPA therapy within an unselected kidney transplant population. This investigation aims to address this knowledge gap and contribute valuable insights for personalized immunosuppressive management following kidney transplantation.

## Materials and methods

### Subjects

This retrospective cohort study was conducted at two local government hospitals, which were the two major kidney transplant centers in Malaysia. Currently, these two hospitals are the only government hospitals that perform kidney transplants. A total of 423 adult KTRs who had undergone their first kidney transplantation between 2011 and 2019 were screened. The management of immunosuppressive therapy was similar in both centers [3]. Those received tacrolimus (Prograf^®^ or Advagraf^®^, Astellas Pharma, Ireland) with MPA and steroids were included. Patients who had undergone re-transplantation (n = 8) were excluded, including those hospitalized for more than 3 months from transplant date (n = 6), incomplete medical records (n = 37) and recipients who were transferred to other hospitals soon after transplant (n = 115). A total of 257 KTRs were finally included.

### Ethical approval

The study protocol was conducted in accordance with the Declaration of Helsinki and approved by the Medical Research Ethics Committee, xxx. The study was registered with the xxx (NMRR ID-22-00054-0GQ (IIR)). The study was also approved by the University’s Research Ethics Committee (PPI/111/8/JEP-2022-431).

### Sample size

The study sample size calculated based on the Krejcie and Morgan’s [11] and factor analysis method [12, 13]. From a total of 432 KTRs at both kidney transplant centers between 2011 and 2019, the calculated sample size required for this study was 205 (at 95% confidence and 5% margin of error). This number was then deliberately exceeded to provide for exclusions and dropouts. In addition, based on 25 variables to be included in the study [12, 13], a sample size of 250 patients was required.

### Data collection

The clinical information was collected retrospectively via chart review from the hospitals’ electronic medical record system into a standardized form. Patients’ names were kept on a password-protected database and were linked only with study-specific identification numbers for this research. This form was divided into four areas: demographic data, clinical information, medication characteristics and outcome information. All patients were followed for 24 months with follow-up points of 1, 3, 6, 12 and 24 months after transplantation.

Demographic data included age, gender, ethnicity and body weight. Clinical information was composed of primary diagnosis, co-morbidities, pre-transplant blood pressure, dialysis modality, dialysis duration and types of transplants. Details of concomitant medication regimen and number of medications taken prior to transplant were recorded.

Clinical outcome included patients’ kidney function, denoted as estimated glomerular filtration rate (eGFR) calculated using the Chronic Kidney Disease Epidemiology Collaboration (CKD-EPI) equation. Body weight, blood pressure reading, tacrolimus daily dose and its corresponding tacrolimus trough level at months 1, 3 and 24, and when switches were made, and reasons immunosuppressives were switched to other alternative immunosuppressive agents were recorded. Other outcome data included the presence of delayed graft function (DGF) defined as the need for dialysis within the first week after transplantation [14], acute rejection diagnosed as BPAR or clinical acute rejection. ADR were noted as written in medical notes by clinicians: CNI toxicity as per biopsy proven result, chronic allograft nephropathy (CAN), PTDM and hypertension treated with medication as diagnosed by the treating nephrologist, hospital admission due to infection, transaminitis, diarrhea, malignancy, CMV and BKV infection, urinary tract infection (UTI) defined by a combination of positive culture with initiation of therapy, acute tubular necrosis (ATN) and leukopenia defined as leukocyte count inferior to 4.0/µL.

### Immunosuppression

Immunosuppressive therapy was divided into two phases: induction therapy and maintenance therapy [3]. In induction phase, methylprednisolone 500 mg was given during the intraoperative period, followed by 250 mg daily starting from day 1 post-transplant operation until patient could ingest oral medication, to which prednisolone 30 mg was prescribed and the dose was tapered down gradually to 20 mg by week 4, then tapered by 2.5 mg every 2 weeks until the dose of 7.5 mg daily was reached at month 3. The dose may be further reduced to 5 mg daily after six-month post-transplant. Either basiliximab (20 mg on operation day and day 4 post operation respectively) or anti-thymocyte globulin induction (1.5 mg/kg/day) ranging from 4 to 7 days was prescribed depending on the subject’s immunological risk. Maintenance therapy involved all patients who received tacrolimus in combination with mycophenolate mofetil or mycophenolate sodium and prednisolone [3]. Initial dose of tacrolimus was 0.1 mg/kg/dose twice daily with trough level adjusted according to target therapeutic level. MPA was prescribed as mycophenolate mofetil at a starting dose of 1g twice daily or mycophenolate sodium at a dose of 1,440 mg/day.

### Statistical analysis

Categorical data were presented as frequencies and percentages. Normally distributed data were presented as means and standard deviations (S.D.s); non-normally distributed data were presented as median (interquartile range). Normality was determined using the Kolmogorov–Smirnov test. To determine which patient’s characteristics were associated with the initial switch from tacrolimus-MPA immunosuppressive therapy to other combination immunosuppressive drug regimen, patients were divided into two groups: switch and maintained in the same drug regimen and a simple and multiple logistic regression analysis were performed. Multiple logistic regression analysis was performed on clinical variables with p < 0.25 in the simple logistic analysis and quantified with odd ratios (OR) and 95% confidence interval (CI). All analyses were carried out using SPSS statistical software (version 23, IBM, SPSS, Chicago, IL, USA). The threshold for statistical significance was set at p < 0.05 (two-sided).

## Results

### Demographic and clinical characteristics

A total of 257 KTRs were included in this study. Patients recruited were mainly male (58.8%, n = 151) with a mean age of 38.9 ± 10.7 years. Malay patients formed the majority of KTRs in this study (60.6%, n = 156), with cadaveric grafts (47.1%, n = 121) being the most common type of kidney transplant. All recipients were maintained with tacrolimus; with 155 (60.3%) subjects on mycophenolate mofetil and 102 (39.7%) had mycophenolate sodium. 17 (6.6%) KTRs were on steroid free regimen for a mean duration of 21.3 ± 14.5 days before steroid was reintroduced to them. Patient demographics, clinical characteristics and medication information are summarized in Table 1.

**TABLE 1:** Baseline patients’ characteristics of the study population (N = 257).

Parameters	n (%) or mean ± SD
Demographic	
Gender	
Male	151 (58.8)
Female	106 (41.2)
Age at transplant	38.9 ± 10.7
Ethnicities	
Malay	156 (60.7)
Chinese	59 (23.0)
Indian	31 (12.1)
Others	11 (4.3)
Clinical	
Primary renal disease	
Autosomal dominant polycystic kidney disease	6 (2.3)
Alports syndrome	5 (1.9)
Analgesic nephropathy	1 (0.4)
Chronic glomerulonephritis	28 (10.9)
Chronic interstitial nephritis	1 (0.4)
Chronic reflux nephropathy	6 (2.3)
Diabetic nephropathy	7 (2.7)
Dysplastic right kidney	1 (0.4)
Focal segmental glomerulonephritis	14 (5.4)
Hypertensive	9 (3.5)
IgA nephropathy	21 (8.2)
Lupus nephritis	5 (1.9)
Obstructive uropathy	3 (1.2)
Polycystic kidney disease	2 (0.8)
Renal calculi disease	1 (0.4)
Tubular dysfunction	1 (0.4)
Unknown	146 (56.8)
BMI, kg/m^2^ ^a^	22.3 ± 3.6
SBP pre-transplant, mmHg^b^	139.4 ± 19.3
DBP pre-transplant, mmHg^b^	84.0 ± 12.1
Number of medications taken prior to transplant^c^	6.8 ± 2.3
Comorbidities	
Hypertension	158 (61.5)
Diabetes mellitus	16 (6.2)
Both hypertension and diabetes mellitus	15 (5.8)
Others	76 (29.6)
Dialysis	251 (97.7)
Preemptive	6 (2.3)
Dialysis modality	
PD	38 (15.1)
HD	180 (71.7)
Mix mode	33 (13.1)
PD then HD	23 (69.7)
HD then PD	10 (30.3)
Duration of dialysis, months^d^	99 ± 78.3
Type of donor	
Living related	91 (35.4)
Kidney source, parent	42 (46.2)
Kidney source, sibling	46 (50.5)
Kidney source, children	1 (1.1)
Kidney source, cousin	2 (2.2)
Living non-related	45 (17.5)
Cadaveric	121 (47.1)
Induction treatment	
IL-2 inhibitor	179 (69.6)
ATG	78 (30.4)
DGF	
Yes	72 (28.0)
No	185 (72.0)
Tacrolimus trough, ng/mL, median (IQR)	
Month 1	8.70 (7.20–10.45)
Month 3	7.40 (6.30–8.50)
Month 24	6.15 (5.20–7.18)
ISA switch	7.45 (6.30–9.57)

Abbreviations: ATG, anti-thymocyte globulin; BMI, body mass index; DBP, diastolic blood pressure; DGF, Delayed graft function; HD, hemodialysis; IL-2, interleukin-2; IQR, interquartile range; PD, peritoneal dialysis; SBP, systolic blood pressure; SD, standard deviation.

^a^ n = 190.

^b^ n = 255.

^c^ n = 191.

^d^ n = 250.

All 257 subjects were followed up for 2 years from transplant date, except when their immunosuppressive agent (ISA) was switched to other ISAs (n = 78, 30.4%), died (n = 2, 0.8%), had graft failure (n = 10, 3.9%) or transferred to other hospitals (n = 53, 20.6%) before the two-year study period.

Two patients died on days 66 and 375 of the follow-up period in this population. Six patients were diagnosed with allograft failure at 1 year, and four patients at 2 years after transplantation, resulting in a cumulative graft survival rate of 96.1% at 2 years.

### Tacrolimus-MPA-prednisolone switch

A total of 81 out of 257 KTRs had their ISAs switched from tacrolimus-MPA immunosuppressive regimen to other combination immunosuppressive regimen at different times due to various reasons. The average time switch of immunosuppressive regimen was 125.8 ± 100.9 days. The median tacrolimus trough concentration before ISA switch was 8.70 ng/mL (IQR 7.20–10.45 ng/mL) and 7.40 ng/mL (IQR 6.30–8.50 ng/mL) at month 1 and 3 following transplantation. At month 24 and ISA switch time, the median tacrolimus trough concentration was 6.15 ng/mL (IQR 5.20–7.18 ng/mL) and 7.45 ng/mL (IQR 6.30–9.57 ng/mL) respectively.

Among those who experienced the switch, 78 (96.3%) KTRs had their initial standard immunosuppression regimen switched to include everolimus, which is an mTOR inhibitor in combination with low-dose tacrolimus. The main reasons observed for the switch include the occurrence of transaminitis (n = 15, 18.5%, Table 2), followed by CMV infection (n = 13, 16.0%), and BK virus infection (n = 10, 12.3%). Other alternative immunosuppressants used were azathioprine (n = 1, 1.2%) and cyclosporine (n = 2, 2.4%).

**TABLE 2:** Reasons for switching from tacrolimus-based to other ISAs (n = 81).

Type of ISA	Reasons for switch	n (%)
mTOR inhibitor	Diarrhea	6 (7.4)
	Leukopenia	9 (11.1)
	BKV infection	10 (12.3)
	CMV infection	13 (16.0)
	BKV and CMV infection	4 (4.9)
	EBV infection	1 (1.2)
	Suboptimal graft function and anemia	7 (8.6)
	Transaminitis	15 (18.5)
	CNI toxicity	4 (4.9)
	High tacrolimus dose required	1 (1.2)
	Neurological effect eg tongue numbness, tremor and dysarthria	4 (4.9)
	Salt losing nephropathy secondary to tacrolimus	1 (1.2)
	PTDM	1 (1.2)
	High variability	1 (1.2)
	Hepatitis	1 (1.2)
Azathioprine	Family planning	1 (1.2)
Cyclosporine	Diabetic control	1 (1.2)
	Neurological effect	1 (1.2)

Abbreviations: BKV, BK virus; CNI, calcineurin inhibitor; CMV, cytomegalovirus; EBV, Epstein-Barr virus; ISAs, immunosuppressive agents; mTOR, mammalian target of rapamycin; PTDM, post-transplant diabetes mellitus.

### Factors affecting initial switch of tacrolimus-MPA-prednisolone

A simple logistic regression demonstrated that ethnicity, hypertension co-morbidity, ATN, CNI toxicity, post-transplant hypertension, CMV and BKV infection were factors affecting the initial switch of immunosuppressive regimen (Table 3). Variables with p-value of <0.25 from simple logistic regression were then included in the multiple logistic regression analysis [15]. It was demonstrated that the Chinese (adjusted Odd Ratios [aOR] 0.408, 95% confidence interval [CI]: 0.187, 0.887) and the Indian patients (aOR 0.179, 95% CI: 0.050, 0.642) were significantly less likely to undergo an immunosuppressive switch as compared to the Malay patients; while patients without post-transplant hypertension (aOR 0.380,95% CI: 0.199, 0.725) and the presence of BKV infection (aOR 7.442, 95% CI: 2.475, 22.379) were found to be determinants of the initial switch to other immunosuppressive regimen after controlling other confounding factors (Table 4). Multicollinearity and interaction terms were checked and not found. Hosmer-Lemeshow test (p = 0.201), classification table (overall correctly classified percentage = 73.4%) and area under the ROC curve (57.4%, 95% CI: 49.6%–65.1%) were applied to check the model fitness.

**TABLE 3:** Factors associated with initial switch from tacrolimus-MPA-prednisolone immunosuppressive therapy to other combination immunosuppressive regimen, N = 257 (Simple model).

Simple model					
Variables (Ref)	B	Unadjusted OR	95% CI		P-value
Demographic					
Male (female)	0.333	1.395	0.810	2.401	0.230
Age, years	−0.008	0.992	0.968	1.017	0.532
Ethnicity (Malay)					
Chinese	−0.751	0.472	0.239	0.931	0.030
Indian	−1.817	0.162	0.047	0.557	0.004
Others	−1.088	0.337	0.070	1.612	0.173
Clinical					
BMI, kg/m^2^	−0.049	0.953	0.874	1.038	0.266
SBP ≥120 mmHg (<120)	0.371	1.450	0.648	3.243	0.366
DBP ≥80 mmHg (<80)	−0.043	0.958	0.547	1.676	0.880
Comorbidities (No)					
Hypertension	0.285	1.329	0.714	2.475	0.037
DM	0.936	2.550	0.152	42.765	0.515
Both hypertension and DM	0.020	1.020	0.287	3.631	0.976
Others	−0.114	0.893	0.327	2.436	0.824
Dialysis (Preemptive)	0.850	2.339	0.269	20.352	0.441
Dialysis modality (HD)					
PD	0.193	1.213	0.578	2.549	0.610
HD then PD	0.336	1.400	0.323	6.067	0.653
PD then HD	0.537	1.711	0.738	3.967	0.211
Dialysis duration (months)	0.002	1.002	0.998	1.005	0.281
Type of donor (Living-related)					
Living non-related	−0.201	0.818	0.369	1.815	0.622
Cadaveric	0.142	1.153	0.644	2.066	0.632
Induction treatment IL2 inhibitor (ATG)	−0.035	0.965	0.545	1.708	0.903
DGF (No)	0.027	1.028	0.573	1.844	0.927
Clinical outcome					
Acute graft rejection (No)	0.174	1.190	0.647	2.189	0.576
ATN (No)	0.740	2.095	1.172	3.7460	0.013
Tacrolimus trough level 1 month (within range)	0.174	1.190	0.683	2.072	0.540
Tacrolimus trough level 3 months (within range)	0.135	1.144	0.647	2.025	0.643
Tacrolimus trough level 24 months (within range)	0.251	1.286	0.358	4.611	0.700
Tacrolimus trough level at switch time, ng/mL	0.033	1.033	0.794	1.346	0.807
ADR (No ADR)					
CNI toxicity	0.959	2.609	1.098	6.196	0.030
PTDM	0.452	1.571	0.694	3.559	0.278
Post-transplant hypertension^a^	−0.560	0.571	0.335	0.973	0.039
Transaminitis	0.359	1.432	0.791	2.591	0.236
Diarrhea	0.109	1.115	0.602	2.064	0.730
CMV	0.796	2.217	1.149	4.278	0.018
BKV	1.618	5.045	1.950	13.049	<0.001
UTI	0.014	1.014	0.550	1.868	0.964
Leukopenia	−0.072	0.930	0.457	1.895	0.842

^a^ Post-transplant hypertension refers to a diagnosis of high blood pressure with the use of anti-hypertensive medications following kidney transplantation [3].

**TABLE 4:** Factors associated with initial switch from tacrolimus-MPA-prednisolone immunosuppressive therapy to other combination immunosuppressive regimen, N = 257 (Multiple model).

Multiple model					
Variables (Ref)	b	Adjusted OR	95% CI		P-value
Male (female)	0.370	1.447	0.779	2.690	0.242
Ethnicity (Malay)					
Chinese	−0.898	0.408	0.187	0.887	0.024
Indian	−1.721	0.179	0.050	0.642	0.008
Others	−0.821	0.440	0.087	2.224	0.321
Comorbid hypertension (No)	0.509	1.664	0.879	3.149	0.118
Dialysis modality (HD)					
PD	0.083	1.087	0.473	2.497	0.845
HD then PD	−0.158	0.854	0.167	4.357	0.849
PD then HD	0.389	1.475	0.570	3.814	0.423
ATN (No)	0.641	1.899	0.955	3.779	0.068
CNI toxicity (No)	0.759	2.136	0.783	5.826	0.138
Post-transplant hypertension (No)	−0.968	0.380	0.199	0.725	0.003
Transaminitis (No)	0.450	1.568	0.789	3.113	0.199
CMV (No)	0.433	1.542	0.716	3.321	0.268
BKV (No)	2.007	7.442	2.475	22.379	<0.001

Abbreviations: ADR, adverse drug reaction; ATN, acute tubular necrosis; BKV, BK virus; CNI, calcineurin inhibitor; CAN, chronic allograft nephropathy, CMV, cytomegalovirus; DGF, delayed graft function; DM, diabetes mellitus; HD, hemodialysis; IL-2, interleukin-2; PD, peritoneal dialysis; PTDM, post-transplant diabetes mellitus; UTI, urinary tract infection.

## Discussion

In Malaysia, KTRs universally receive initial and maintenance immunosuppression with a tacrolimus-MPA-prednisolone triple therapy regimen, as endorsed by the Kidney Disease: Improving Global Outcomes (KDIGO) Transplant Workgroup (2009). This approach is well-supported by its established efficacy in reducing acute rejection episodes and promoting graft function [4]. Our current study reflects this success, demonstrating a high overall graft survival rate of 97.7% and 96.1% at one and 2 years, respectively, which aligns with previous findings [16]. However, despite the initial effectiveness of the tacrolimus regimen, a significant proportion of patients required a switch to alternative immunosuppressants, with the average switch occurring around 17 weeks, typically within the first 10–16 weeks post-transplant. The majority of kidney transplant recipients transitioned from the initial tacrolimus–MPA–prednisolone regimen to a combination of everolimus and low-dose tacrolimus, reflecting similar findings by Taber et al., who reported a comparable switch timeframe [7]. Therefore, elucidating the associated factors that prompt this early regimen switch to mostly everolimus combined with low-dose tacrolimus, holds significant value for nephrologists, pharmacists and other healthcare professionals, allowing them to optimize post-transplant management strategies for KTRs.

The occurrence of transaminitis and infections were the primary reasons for switching immunosuppressants, with BKV as a significant factor in influencing the switch of immunosuppressants. Everolimus combined with low-dose tacrolimus emerged as the preferred alternative. Studies by Saliba et al. support this approach, demonstrating no significant difference in liver enzymes after a year compared to traditional regimens [17]. Additionally, Pascual et al. reported a lower incidence of abnormal liver function tests with everolimus compared to MPA, further justifying the switch [18]. The observed link between immunosuppression and viral infections, including CMV and BKV, aligns with findings from Vanichanan et al. who reported a higher prevalence in Asia [19]. This association is likely due to the combined effect of potent immunosuppression and high-risk transplants (e.g., blood group incompatibility) requiring aggressive preconditioning to minimize rejection [20]. These complications observed stem from the inherent nature of tacrolimus-based immunosuppression independent of drug concentration, where the immunosuppressive effect compromises the immune system’s ability to control viral replication [8]. This was further confirmed by our results which showed no significant association between tacrolimus trough levels and ISA switch. In contrast, everolimus exerts less T-cell suppression compared to tacrolimus besides having shown to have some anti-viral properties against CMV and BKV [20]. Therefore, simply reducing immunosuppression may not be suitable. Instead, as suggested by Tan et al., switching to mTOR inhibitors, specifically everolimus with low-dose tacrolimus, offers a safer alternative, potentially balancing immunological risk minimization with mitigation of ADR associated with traditional regimens like MPA [22], offering a dual benefit in managing KTRs.

Despite this, it is important to understand that switching to everolimus with low-dose tacrolimus or other combination immunosuppression may lead to undesired outcomes such as increased rejection risk or unresolved viral load, which was not investigated in the present study. A balanced approach in modifying immunosuppressive therapy is therefore advocated. While reduced immunosuppression may help in managing BKV and CMV infections, it may compromise graft survival due to heightened immune activation [23]. These conflicting outcomes mandate the need for individualized patient management especially among patients with high immunological risk, where switching of ISA should be accompanied by close monitoring of viral load, immune risk profiling and careful timing of intervention when needed.

This study also identified ethnicity as a significant factor influencing immunosuppressant switches among KTRs, with Malays demonstrating a higher propensity for regimen changes compared to other ethnicities. While prior research suggests immunological differences potentially contribute to racial variations in graft survival, existing literature largely focuses on Caucasian populations in Western countries [24]. Limited data exists regarding the specific susceptibility of Malays to early switches from the tacrolimus-MPA-prednisolone regimen. However, one study by Mastuki reported a higher prevalence of CMV infection in Malays, possibly prompting adjustments in immunosuppression [25]. On the other hand, the impact of ethnicity on response variation in immunosuppressive therapy may be attributable to genetic variations [26]. Variations in the pharmacokinetic profile of immunosuppressants are attributed to inter-individual variations in the functional activity of metabolizing enzymes and drug transporters, which are governed by the differences in their pharmacogenetic properties depending on ethnic groups [26]. One’s ethnicity may influence one’s health or disease condition, for example the Asian Americans were reported to have significantly higher risk of cardiovascular diseases [27]. Since there is currently little data on the prediction of ADRs or efficacy of immunosuppressants due to genetic variation in association with different ethnic backgrounds, more research is needed with the understanding that ethnic considerations might provide us with valuable information for optimizing individualized immunosuppressive therapy following kidney transplantation. Given that Malays comprise the largest ethnic group in Malaysia, further investigation into the factors governing their response to everolimus, a potentially cost-prohibitive therapy [10], is warranted. While the observed association between Malay ethnicity and initial regimen switches requires further exploration, it holds promise as a potential marker for identifying patients at higher risk for early switches, thereby facilitating closer monitoring and potentially optimizing post-transplant management strategies.

Factors beyond traditional ADRs influenced the decision to switch immunosuppressive regimens in the current KTRs. Notably, the absence of post-transplant hypertension emerged as a significant predictor for switching away from the tacrolimus-MPA-prednisolone regimen. This finding is intriguing as some studies suggest a potential link between everolimus, a common alternative therapy, and an increased prevalence of hypertension [28]. While the exact mechanisms remain unclear, one study reported a rise in arterial hypertension from 49% to 65.9% following conversion to everolimus [28]. However, it’s important to note that other studies haven’t observed a significant impact on mean blood pressure [29]. These contrasting findings highlight the need for further research into the relationship between everolimus and blood pressure. Regardless, our study underscores the importance of vigilant blood pressure monitoring, particularly for patients transitioning to everolimus-based regimens, to minimize the potential for cardiovascular complications [29]. This unexpected association between hypertension and immunosuppressive switch warrants further investigation to optimize post-transplant management strategies for KTRs.

### Study limitations

This study’s retrospective design and reliance on electronic health record reviews introduce inherent limitations. Potential inaccuracies or inconsistencies in data collection and recording during the study period cannot be ruled out. Additionally, the study is limited by the quality of data originally captured for clinical purposes, which may not have been specifically designed to address the research questions posed here. Furthermore, even documented ADRs might not be entirely captured if not readily apparent or attributed to immunosuppressive medications. Additionally, the observed benefit could be attributed to drug interactions between unaccounted-for medications and the immunosuppressants, rather than solely the immunosuppressants’ effects. Minor inter-center variations in clinical practice, particularly in infection screening and management could introduce variability in patient outcomes. Also, genetic factors vary significantly across different ethnic groups and may potentially influence the observed outcomes. While this study did not fully explore genetic contributions, it is crucial to acknowledge their potential impact. Finally, the generalizability of our findings may be limited, as the study population was restricted to two centers in Malaysia.

## Conclusion

In conclusion, this study sheds light on factors beyond traditional ADR that influence the decision to switch immunosuppressive regimens in KTRs. Our findings suggest that Malay ethnicity, the absence of post-transplant hypertension, and BKV infection are significant predictors for switching from the initial tacrolimus-MPA regimen. By identifying these factors healthcare professionals can implement closer monitoring and potentially explore alternative therapies like everolimus with greater discernment. While existing data indicate that such switches typically occur within the first 10–16 weeks post-transplant following viremia, clear guidelines on optimal timing and its impact on graft outcomes remain limited. Ultimately, this study underscores the importance of a nuanced approach to immunosuppression in kidney transplantation. This shift away from a one-size-fits-all model and towards personalized regimens that balance efficacy with patient-specific considerations has the potential to improve long-term patient outcomes. By incorporating these findings into treatment plans, healthcare professionals can create individualized immunosuppressive strategies that minimize the risk of complications while ensuring adequate graft function. This tailored approach holds promise for not only improving patient survival rates but also enhancing their overall cost and quality of life following transplantation.

## Data Availability

The original contributions presented in the study are included in the article/supplementary material, further inquiries can be directed to the corresponding author.
